# Vertical Graphenes Grown on a Flexible Graphite Paper as an All-Carbon Current Collector towards Stable Li Deposition

**DOI:** 10.34133/2020/7163948

**Published:** 2020-07-11

**Authors:** Zhijia Huang, Debin Kong, Yunbo Zhang, Yaqian Deng, Guangmin Zhou, Chen Zhang, Feiyu Kang, Wei Lv, Quan-Hong Yang

**Affiliations:** ^1^Shenzhen Geim Graphene Center (SGC), Tsinghua-Berkeley Shenzhen Institute (TBSI), Tsinghua Shenzhen International Graduate School, Tsinghua University, Shenzhen 518055, China; ^2^CAS Key Laboratory of Nanosystem and Hierarchical Fabrication, CAS Center for Excellence in Nanoscience, National Center for Nanoscience and Technology, Beijing 100190, China; ^3^Shenzhen Key Laboratory for Graphene-based Materials, Engineering Laboratory for Functionalized Carbon Materials, Tsinghua Shenzhen International Graduate School, Tsinghua University, Shenzhen 518055, China; ^4^Nanoyang Group, State Key Laboratory of Chemical Engineering, School of Chemical Engineering and Technology, Tianjin University, Tianjin 300072, China

## Abstract

Lithium (Li) metal has been regarded as one of the most promising anode materials to meet the urgent requirements for the next-generation high-energy density batteries. However, the practical use of lithium metal anode is hindered by the uncontrolled growth of Li dendrites, resulting in poor cycling stability and severe safety issues. Herein, vertical graphene (VG) film grown on graphite paper (GP) as an all-carbon current collector was utilized to regulate the uniform Li nucleation and suppress the growth of dendrites. The high surface area VG grown on GP not only reduces the local current density to the uniform electric field but also allows fast ion transport to homogenize the ion gradients, thus regulating the Li deposition to suppress the dendrite growth. The Li deposition can be further guided with the lithiation reaction between graphite paper and Li metal, which helps to increase lithiophilicity and reduce the Li nucleation barrier as well as the overpotential. As a result, the VG film-based anode demonstrates a stable cycling performance at a current density higher than 5 mA cm^−2^ in half cells and a small hysteresis of 50 mV at 1 mA cm^−2^ in symmetric cells. This work provides an efficient strategy for the rational design of highly stable Li metal anodes.

## 1. Introduction

The commercial lithium-ion batteries cannot meet the demand for the fast development of electric vehicles and electronic devices due to their low energy density [1, 2]. In order to further improve the energy density, the lithium metal has been considered as the most promising anode material for the next-generation high-energy density batteries with advantages of ultrahigh theoretical capacity (3860 mAh g^−1^), low density (0.59 g cm^−3^), and the lowest reduction potential (-3.04 V versus the standard hydrogen electrode) [3]. However, the safety hazards and low Coulombic efficiency (CE) of Li metal anode (LMA) triggered by dendrite growth and continuous side reactions need to be addressed before its practical use [4–8]. The growth of Li dendrites is caused by nonuniform Li nucleation and growth. In addition, the unstable solid electrolyte interphase (SEI) on the Li surface cracks due to the volume changes and reforms during the Li plating/stripping processes which continuously consumes the Li-ions and electrolytes, resulting in fast capacity fading and low Coulombic efficiency [9–11]. All these drawbacks impede the practical applications of LMA.

To circumvent these issues, tremendous efforts have been adopted to suppress Li dendrite growth and enhance the electrochemical performance of LMA. One strategy is to stabilize the Li metal surface by artificial SEI or Li-based alloys [12–18]. However, how to maintain these layers stable when experiencing the large volume variation of Li under high current density as well as high capacity is a great challenge. Recently, using the 3D conductive frameworks as Li hosts has been proved as an effective way to suppress Li dendrite growth and accommodate the volume change [19–23]. The increased electroactive surface area can reduce the local current density and homogenize Li^+^ ion flux. 3D porous metallic (e.g., Cu or Ni) current collectors and lithiophilic surface modification of commercial metallic frameworks have been shown their advantages in suppressing Li dendrite growth and enabling uniform Li deposition [24–29]. Compared to the porous metals, porous carbon matrices have distinct advantages of lightweight, high electric conductivity, and excellent flexibility as well as stability. In previous studies, different types of carbon-based materials have been used as stable Li hosts [30–40]. However, the poor affinity of most of these carbon skeletons with Li causes a large nucleation overpotential and cannot realize uniform Li nucleation. Moreover, the mass transport behavior during Li plating/stripping is limited due to the high tortuosity of these disordered porous structures, which further leads to the nonuniform disposition.

**Figure 1:**
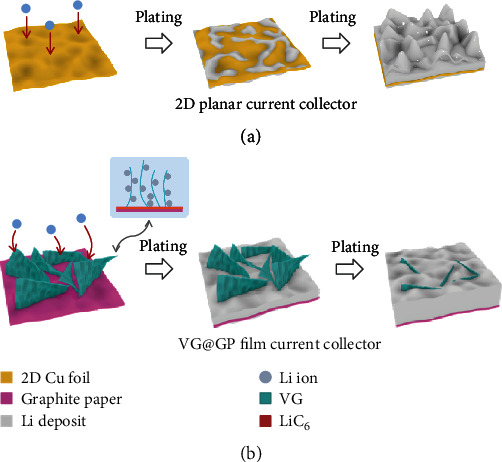
Schematic view of the Li deposition behavior on (a) 2D planar current collector and (b) VG@GP current collector.

To solve the above problems, herein, we design a hybrid carbon structure that the vertical graphene (VG) array with a height less than 2 *μ*m grown on graphite paper (GP) (VG@GP) to enable uniform Li nucleation and deposition. In this structure, the VG structure provides a comprehensive contact with the electrolyte through their large surface area and thus effectively reduces the local current density. Particularly, the perpendicular open structure enables the uniformly distributed electric field and fast ion diffusion to decrease the polarization induced by the formation of ion gradients. At the same time, the GP paper becomes a lithiophilic substrate and current collector after the initial reaction with Li to form LiC_6_, which largely reduces the Li nucleation overpotential and thus guides the Li deposition from the bottom.  Figure 1 shows the schematic view of Li deposition behavior on the VG@GP film in comparison with the deposition on the ordinary substrate (e.g., Cu foil). Note that the weight of VG on the GP is negligible. The density of VG@GP film is about 1.54 g cm^−3^, which is quite lower than that of Cu foil (6.05 g cm^−3^), showing its ultralight nature. With these benefits, the uniform Li deposition is achieved where the growth of Li dendrites is effectively suppressed. The Li anode using VG@GP film shows an excellent cycling performance with a high CE of 95.8% over 100 cycles at a high current density of 2 mA cm^−2^. The symmetric cells also exhibit stable cycling performance with a low overpotential of 50 mV over 400 h at 1 mA cm^−2^ with a capacity of 1 mAh cm^−2^. Moreover, stable cycling performance with high CE is obtained in the full cells by using VG@GP Li anode.

## 2. Results and Discussion

**Figure 2:**
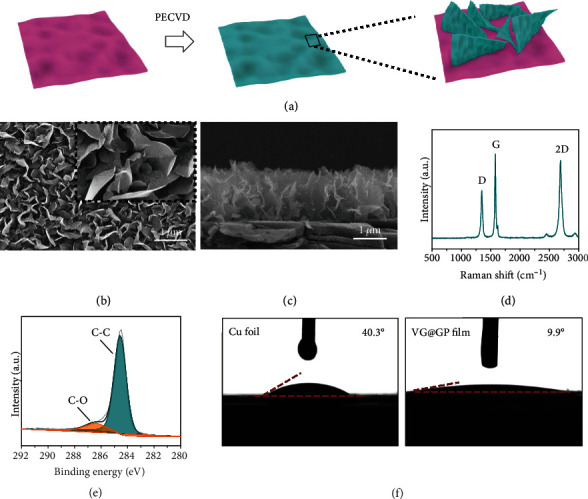
(a) Schematic representation of the VG@GP fabrication process. (b) Top-view SEM image of the surface morphology of VG@GP. (c) The cross-sectional view SEM image of VG@GP. (d) Raman spectra of VG@GP. (e) XPS of the C1s spectrum of VG@GP. (f) The contact angles of electrolyte on Cu foil and VG@GP.

Figure 2(a) shows the schematic view of the fabrication process of VG@GP film by a plasma-enhanced chemical vapor deposition (PECVD) using CH_4_ as a carbon source (Yick Xin Technology Development Ltd. Co. (Shenzhen, China)). As shown in Figure S1, the vertical graphene can be deposited on the GP substrate with a diameter of 20 cm, and the average mass loading of VG on GP is less than 0.02 mg cm^−2^, which is light and does not introduce extra weight to the batteries. As shown in Figure 2(b), the surface of GP is fully covered by uniform vertical aligned graphene, and they interconnect with each other, and the average interspace between them is around 200 nm. From the cross-sectional view in Figure 2(c), the average height of VG is less than 2 *μ*m, and they directly attach to the GP substrate, which helps to enhance the structural stability and reduce the contact resistance between them. With such a unique structure, the VG helps to reduce the local current density and provides abundant nucleation sites. More importantly, the highly ordered vertical structure leads to the uniformly distributed electric field and Li-ion distribution on the electrode surface and ensures the fast Li^+^ ion diffusion. Moreover, the 3D structure also decreases the local current density on the electrode surface. All these structure characters guarantee stable and uniform deposition. In Raman spectra (Figure 2(d)), the strong intensity of G band peak indicates the formation of graphitized structure with high crystallinity, and the similar intensity of 2D peak to that of G band peak suggests the few-layer graphene on the GP surface [41]. The intensity ratio of D band to G band, *I*_D_/*I*_G_, is 0.58, showing the existence of abundant defects and edges. The plenty of edges and defects in VG can act as lithiophilic sites to reduce the Li nucleation energy barrier [42, 43]. Meanwhile, Li/C compound can be formed in the edge-rich multilayer graphene due to Li intercalation at a relatively low potential, further increasing the lithiophilicity of whole electrode [44]. The surface chemistry of the VG@GP film is analyzed by X-ray photoelectron spectroscopy (XPS). The atomic concentrations of C and O elements are about 98.0 and 2.0%, respectively. These structure characters and surface chemistry ensure the fast electron transfer for the VG@GP host for Li deposition. In addition, the high-resolution spectrum of C 1s (Figure 2(e)) can be deconvoluted into two peaks located at 284.5 and 286.5 eV, which are assigned to C-C and C-O species. The oxygen functional groups help increase the wetting ability of VG structure by the electrolyte. Figure 2(f) illustrates the wetting ability of the Cu foil and VG@GP film, which shows the much smaller contact angle of electrolyte on VG@GP film (9.9°) than that on planar Cu foil (40.3°), indicating a better wetting ability for VG@GP film due to the vertical structure, which further ensures fast Li^+^ ion transport.

**Figure 3:**
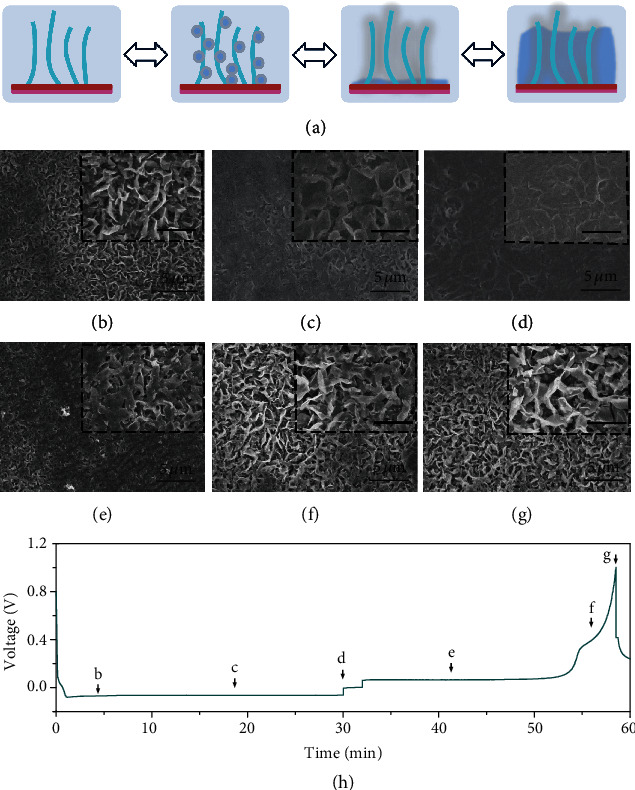
Illustration of Li plating/stripping behavior on the VG@GP. (a) Schematic showing the Li plating behavior on the VG@GP. SEM images of VG@GP after plating (b) 0.05 mAh cm^−2^, (c) 0.3 mAh cm^−2^, and (d) 0.5 mAh cm^−2^ of Li and after stripping (e) 0.2 mAh cm^−2^, (f) 0.45 mAh cm^−2^, and (g) 0.5 mAh cm^−2^ of Li from VG@GP. Li plating/stripping states (b–g) are marked in the (h) galvanostatic discharge/charge voltage profile obtained at 1 mA cm^−2^. The inset scale is 2 *μ*m.

The Li plating/stripping behaviors on VG@GP at different stages were explored on the Li||VG@GP half cell with areal capacities ranging from 0.05 to 0.5 mAh cm^−2^ at a current density of 1 mA cm^−2^.  Figure 3(a) shows the schematic diagrams of Li deposition behavior on the VG@GP. The GP can spontaneously form LiC_6_ compound with Li due to the intercalation reaction of Li into the layer structure of the graphite during the discharge process at 0.1-0.01 V (Figure 3(h)) [45], which increases the lithiophilicity of the substrate and enables uniform Li plating/stripping at low potential. The formed LiC_6_ layer has excellent lithiophilicity to decrease the Li nucleation barrier and increase the nucleation sites, which helps to regulate uniform Li nucleation and growth [46]. The XRD patterns of graphite paper before and after initial Li plating are shown in Figure S2, confirming the Li intercalation into graphite [47–49]. The Li^+^ ions are distributed uniformly inside the VG film and with further plating process, Li is deposited into the channels between the graphene sheets, and with the increase of Li deposition areal capacity, the channels are gradually filled with the Li from inside to outside. As the capacity further increased, the Li fully covers the surface of VG@GP with a dendrite-free morphology. The above Li metal plating/stripping processes were confirmed by the ex-situ SEM images. Figures 3(b)–3(d) show the top-view SEM images of the morphology changes during Li plating processes on the VG@GP film. The VG@GP is firstly lithiated due to the reaction between GP and Li, which forms LiC_6_ enhancing the Li affinity and lowering the nucleation overpotential. After plating of 0.05 mAh cm^−2^ Li, there is no obvious surface morphology change except for the uniformly decorated Li deposition between VG channels (Figure 3(b)). When the Li deposition capacity increases to 0.3 mAh cm^−2^, the open channels and the interspaces are partially filled with the Li deposits (Figure 3(c)). With a further increase of the Li plating capacity to 0.5 mAh cm^−2^, the VG@GP matrix is fully covered by Li deposits with an even surface, indicating the uniform Li deposition (Figure 3(d)). The deposited Li metal can also be reversibly stripped from VG@GP film. Figures 3(e)–3(g) show the surface morphologies of the Li-deposited VG@GP after stripping. The Li metal is gradually stripped from the 3D matrix with the reappearance of the vertical structure, and after charging to 1 V (Figure 3(h)), almost all the Li pieces are stripped completely from the matrix. Most interestingly, the 3D vertical structure remains stable after Li stripping, demonstrating its excellent structural stability.

**Figure 4:**
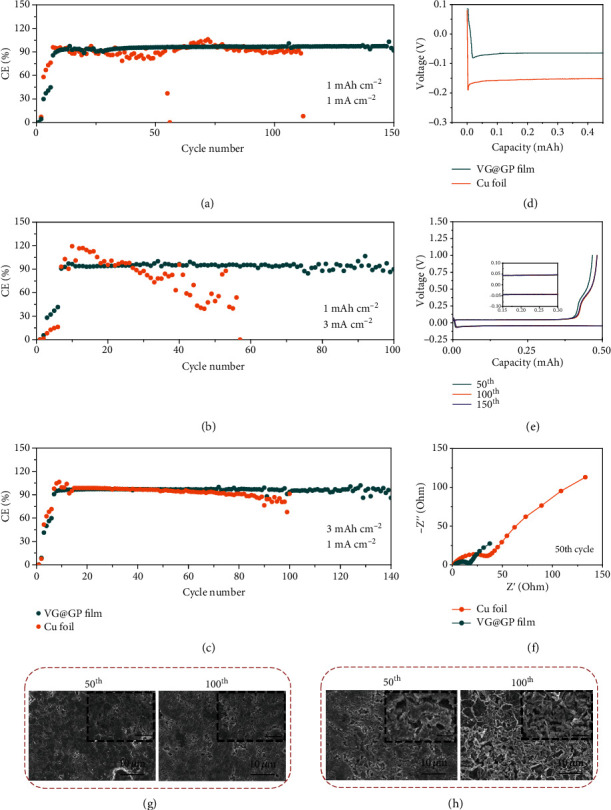
Cycling performance of VG@GP and Cu foil electrodes: (a) at 1 mA cm^−2^ with a total capacity of 1 mAh cm^−2^, (b) at 3 mA cm^−2^ with a total capacity of 1 mAh cm^−2^, and (c) at 1 mA cm^−2^ with a total capacity of 3 mAh cm^−2^. (d) The voltage–capacity curves during Li nucleation at 1 mA cm^−2^. (e) Voltage profiles of VG@GP electrode at 1 mA cm^−2^ and 0.5 mAh cm^−2^. (f) The electrochemical impedance spectra (EIS) of the electrodes after 50 cycles. SEM images of the top surface of Li deposited after 50 and 100 cycles at a current density of 1 mA cm^−2^ with a total capacity of 1 mAh cm^−2^ on VG@GP (g) and Cu foil (h). The inset scale bar is 2.5 *μ*m.

The Coulombic efficiency (CE) and long-term electrochemical stability were evaluated in a half-cell configuration consisting of metallic Li as counter electrode coupled with working electrodes (VG@GP, GP, and Cu foil) and the CE of each cycle was determined by the ratio of the amount of stripped Li to that of as-plated Li. Figures S3–5 and Figures 4(a)–4(c) show the CEs of these electrodes after long cycling with different current densities and deposited capacities. As shown in Figure S3, at a current density of 1 mA cm^−2^ with the area capacity of 0.5 mAh cm^−2^, the CE of Cu foil drops rapidly in the initial several cycles and then fluctuates during long cycling. The unstable cycling performance and low CE values are related to nonuniform Li deposition and unstable SEI formation that are continuously consuming of both Li and electrolytes. In contrast, the VG@GP film electrode maintains stable with a high average CE value of 95.8% after 200 cycles, demonstrating its superior cycling stability. With a high capacity of 1 mAh cm^−2^, the VG@GP also exhibits a stable and high CE of 97.1% over 150 cycles, while the CE of Cu foil becomes unstable after several cycles (Figure 4(a)). When the current density increases to 2 mA cm^−2^ and 3 mA cm^−2^, the CE of VG@GP still remains stable and achieves relatively high CEs after 100 cycles (Figure S4) (Figure 4(b)). Even at a high current density of 5 mA cm^−2^, the VG@GP also shows a relatively stable cycling performance compared to that of Cu foil (Figure S5). The electrochemical performance under high capacity (3 mAh cm^−2^) was also examined, where a stable cycling performance of VG@GP film over 140 cycles can be obtained (Figure 4(c)). The cycling performance of the bare GP substrate as the current collector was also tested. As shown in Figures S6–8, the bare GP electrodes show poor cycling stability with low CE with a capacity of 1 mAh cm^−2^ at different current densities from 1 to 3 mA cm^−2^. Although the bare GP electrode forms LiC_6_ during the initial plating process, it cannot effectively regulate the following Li growth without the surface VG structure. Thus, the excellent cycling performance of VG@GP electrode could be interpreted as a synergistic effect of the GP substrate and unique VG structure, which not only ensures uniform Li nucleation and growth but also helps to even the electric field and homogenize Li-ion flux.

The voltage-capacity profiles further demonstrate Li nucleation behaviors. It can be seen that the VG@GP exhibits a nucleation overpotential of 16.7 mV, much smaller than that of the Cu foil (38.4 mV) (Figure 4(d)). The nucleation overpotentials of these two electrodes at different current densities are further examined. The Cu foil electrode exhibits large Li nucleation overpotential of 55.2, 59.9, 71.9, and 95.8 mV, respectively, at current densities of 1, 2, 3, and 5 mA cm^−2^, but VG@GP shows remarkably reduced overpotentials of 25.4, 27.6, 35.9, and 41.5 mV (Figure S9). Figure S10 shows the detailed discharge-charge profiles of Li plating/stripping on VG@GP. It can be seen that the charge/discharge profiles exhibit a typical lithiation behavior at the initial discharging process before Li plating and a Li deintercalation stage at the end of charging during Li stripping. The lithiation process is further confirmed by CV test. The reduction peak in CV profiles indicates the intercalation of Li-ions into GP and VG (Figure S11), which forms LiC_6_ to enhance the Li affinity and thus provides lithiophilic sites to lower the overpotential and promotes uniform Li nucleation. Figure 4(e) shows the voltage profiles of Li plating/stripping processes in VG@GP after long cycling at a current density of 1 mA cm^−2^ with a capacity of 0.5 mAh cm^−2^. The charge/discharge profiles of VG@GP show no obvious changes even after 150 cycles. However, the voltage profiles of Cu foil are less stable after long cycling, indicating a large amount of irreversible capacity loss (Figure S12). The changes of voltage hysteresis of VG@GP are shown in the inset of Figure 4(e), which decrease and then remain stable at ~90 mV after 150 cycles. On the contrary, the voltage hysteresis of planar Cu foil decreases and then increases after 100 cycles, exhibiting larger voltage hysteresis of 160 mV. The stable charge/discharge profiles with a small overpotential of VG@GP film indicate excellent Li plating/stripping behavior and lower interfacial resistance. Figure 4(f) shows the electrochemical impedance spectra (EIS) of the electrodes after 50 cycles. The charge transfer resistance and interfacial resistance can be represented by the semicircle at the high-frequency region in Nyquist plots. As shown in Figure 4(f), the resistance of VG@GP is much smaller in comparison with that of Cu foil after 50 cycles, revealing the formation of a much more robust SEI and faster Li deposition/dissolution kinetics, benefiting for uniform Li deposition and excellent electrochemical properties. The evolution of SEI during cycling was further investigated by X-ray photoelectron spectroscopy (XPS). Figure S13 shows the profiles of C 1s and F 1s spectra of VG@GP and Cu foil electrodes after 10 cycles. The main components in the SEI film formed on the VG@GP are C-C, C-O and C-F groups [24], while for the Cu foil, the SEI layer mainly contains C-C and C-O groups. The F 1s spectra of both electrodes also show an obvious distinction. A strong peak of Li-F was detected from the surface of VG@GP, which indicates an increase in fluorinated compound of LiF [50]. The enrichment of fluorinated compound such as LiF helps to form a stable SEI film to allow uniform Li plating and stripping, thus suppressing Li dendrite growth and improving the cycling performance.

The morphology of Li metal deposition on different current collectors after long cycling was also investigated to confirm the merits of VG@GP. Figure S14 shows the surface morphology of Li plating after multiple cycles at a current density of 1 mA cm^−2^ with a capacity of 0.5 mAh cm^−2^. The Li deposited on VG@GP displays a smooth and dense surface without detectable dendrites or mossy Li after 50 cycles. After 100 cycles, the morphology still shows a flat surface, demonstrating the uniform Li deposition and high cycling stability (Figure S14 a-b). On the contrary (Figure S14 c-d), the Cu foil exhibits a rough surface with lots of cracks and mossy Li after 50 cycles and becomes much worse when the cycle number increased to 100 cycles. As increasing the areal capacity to 1 mAh cm^−2^, the same trend can be seen, where the surface of VG@GP shows no dendrite (Figures 4(g) and 4(h)). Figures S15 and 16 show the surface morphology of Li deposited on bare GP electrodes. It can be seen that the GP electrodes displays a nonuniform Li deposition with cracks and significant dead Li formation after long cycling. The morphologies under high current density (3 mA cm^−2^) and high capacity (3 mAh cm^−2^) were also examined, where the uniform and dendrite-free surface can be maintained for VG@GP electrode, further indicating the advantages of such structure on guiding Li deposition behavior (Figures S17-18). The vertical open channels not only help to reduce the local current density and regulate the electric field and Li-ion flux but also enable fast Li-ion diffusion on the electrode surface. In addition, the enhanced Li affinity also promotes uniform Li nucleation and growth. The VG@GP also shows good structural stability under the pressure during cell assembly without destroying the vertical structure. Under the pressure of cell assembly, the VG structure with a higher height of 5 *μ*m can still be maintained even (Figure S19). In addition, the VG shows strong adhesion ability to the GP substrate and can maintain stability in water with a stirring speed of 500 revolutions per minute (data provided by Yick Xin Technology Development Ltd). However, the structure of 3D current collectors such as Ni foam or Cu foam cannot be well maintained with the pressure of cell assembly, as seen in Figure S20. The dense structure after pressing can reduce the exposed surface area, and as a result, the CE of the pressed Ni foam drops after 40 cycles under high current density (3 mA cm^−2^) (Figure S21).

**Figure 5:**
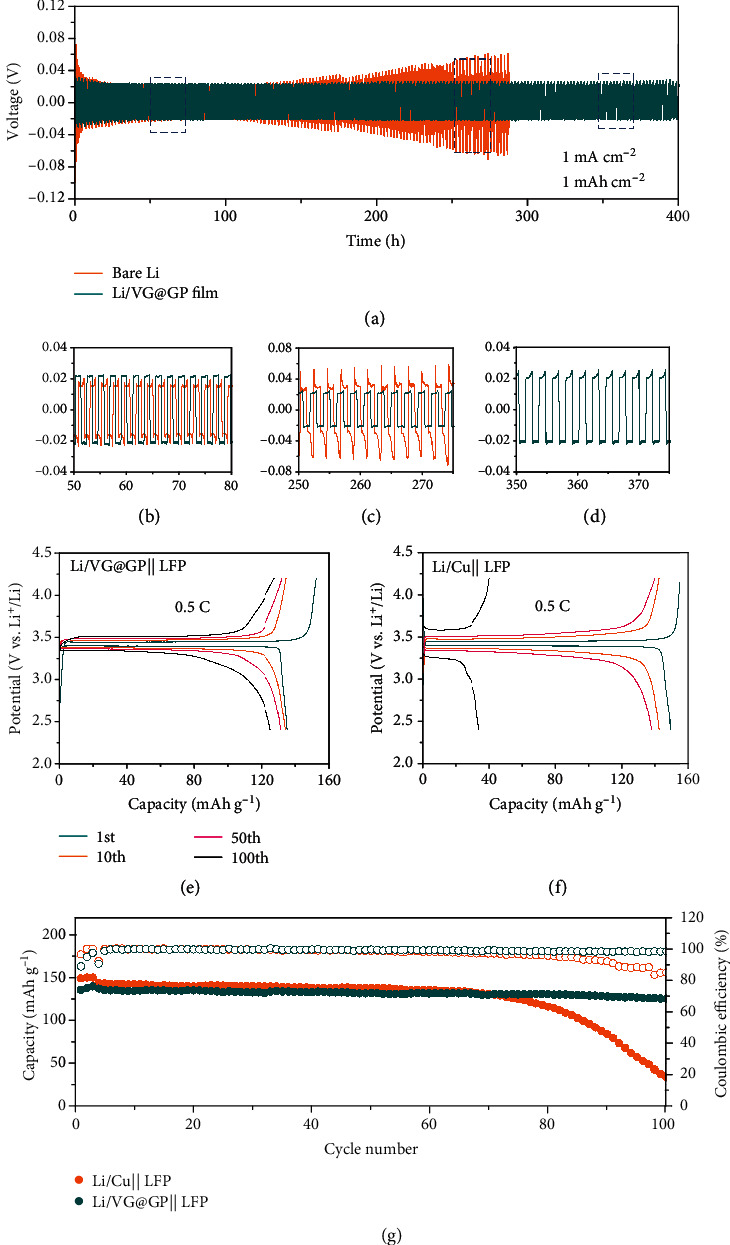
(a) Voltage profiles of Li plating/stripping of symmetric cells (Li foil and Li/VG@GP electrodes) and (b–d) the detailed voltage profiles from 50 h to 75 h, 250 h to 275 h, and 350 h to 375 h. Voltage profiles of (e) the Li/VG@GP‖LFP full cell and (f) the Li/Cu‖LFP full cell. (g) Cycling performance of Li/VG@GP‖LFP and Li/Cu‖LFP full cells at 0.5 C.

The symmetric cells of bare Li and Li-deposited VG@GP (Li/VG@GP) electrodes were assembled to investigate the long-term cycling stability of Li anode.  Figure 5(a) shows the voltage profiles of bare Li and Li/VG@GP at a current density of 1 mA cm^−2^ with a capacity of 1 mAh cm^−2^. The overpotential of bare Li maintains stable in the initial 150 h and then sharply increases after 250 h, showing significant voltage fluctuations with a large overpotential of ca. 150 mV. The increase in hysteresis and unstable voltage profiles of bare Li is a result of the unstable interface and the formation of mossy and dead Li after repeated plating/stripping. Compared to the bare Li, the Li/VG@GP anode maintains stable overpotential after 400 h with a much lower overpotential of ca. 50 mV. The enlarged voltage profiles in Figures 5(b)–5(d) also indicate a relatively flat Li plating/stripping plateau for Li/VG@GP anode. Even with a high current density of 10 mA cm^−2^, Li/VG@GP still exhibited low overpotential (Figure S22), implying stable interfacial properties and effective suppression of dendrite growth at high current densities.

To demonstrate the potential use of such VG@GP current collector in practical applications, full cells were assembled with LiFePO_4_ (LFP) as a cathode material and the VG@GP or Cu foil plated with 5 mAh cm^−2^ Li as the anode. Figures 5(e) and 5(f) present the voltage profiles of full cells with Li/VG@GP|||LFP and Li/Cu||LFP at 0.5 C after cycling. The Li/VG@GP||LFP cell exhibits a lower polarization between discharge and charge profiles compared with that of Li/Cu||LFP cell, especially after long cycling. The cycling performance of both full cells at 0.5 C is illustrated in Figure 5(g). The Li/VG@GP||LFP cell delivers a stable cycling performance with a reversible capacity of 125.4 mAh g^−1^ and a high CE of 98.16% after 100 cycles, which is nearly 92.7% capacity retention of the initial capacity (135.3 mAh g^−1^). While for the Li/Cu||LFP cell, the capacity rapidly decays after 70 cycles, showing its significant capacity fading. The long cycling performance was also examined at 0.5 C (Figure S23). The Li/VG@GP||LFP delivers a high capacity retention of 86.6% after 300 cycles, indicating a good cycling stability. The full cells with a higher cathode loading of 20 mg cm^−2^ (3.1 mAh cm^−2^) with negative/positive capacity ratio (*N*/*P* ratio) of 2.6 at 0.3 C were further tested (Figure S24). The specific capacity of Li/Cu||LFP full cell fades rapidly from 141.1 to 87.8 mAh g^−1^ after 50 cycles. In comparison, the Li/VG@GP||LFP full cell shows a higher initial specific capacity of 162.7 mAh g^−1^ and can maintain at 115.4 mAh g^−1^ after 100 cycles, showing much better cycling stability. This should be mainly ascribed to the uniform Li deposition and the stable interface with the help of lithiophilic substrate and unique VG structure. With a lower *N*/*P* ratio of 1.3, the Li/VG@GP||LFP cell exhibits similar cycling stability, but the fluctuation appears during cycling. The higher area capacity induces a higher current density and an increased fraction of Li metal reacting in each cycle, which may lead to the fast Li degradation and depletion with a low *N*/*P* ratio [36]. Overall, the excellent cycling performance of the full cells with Li/VG@GP anode demonstrates the feasibility of such material in the practical use of Li metal batteries.

## 3. Conclusion

We demonstrate an all-carbon current collector, which is a graphite paper with vertical graphenes grown on its surface, realizing the stable Li deposition. Compared with the other 3D porous collectors, such VG@GP film shows the advantages of low weight and small volume in the battery, which not only ensures the structural stability in the battery assembly process but also maintains the high energy density of the battery. In the VG@GP, the vertically aligned graphene structure on the surface reduces the local current density, regulates the uniform electric field and Li^+^ ion distribution, and guarantees fast ion transfer on the electrode surface, and at the same time, the GP is lithiated at the beginning which increases its lithiophilicity, guiding the Li deposition from the bottom and ensuring the high space utilization of the vertical structure. As a result, the 3D VG@GP electrode exhibits a stable cycling performance at a high current density (even higher than 5 mA cm^−2^) in half cells. A long cycle life with small hysteresis in symmetric cells indicates its stable plating/stripping behavior. Moreover, the full cells that coupled with LFP cathode also reveal its excellent cycling stability and the potential in practical uses. Our study affords an efficient strategy to direct Li nucleation and growth and shows that the rational design of carbon-based materials is of great importance for advanced Li metal anode in high-energy Li metal batteries.

## 4. Experimental Section

### 4.1. Material

Vertical graphene (VG) thin film materials were provided by Yick Xin Technology Development Ltd. Co. (Shenzhen, China). The VG was deposited on graphite paper (GP) in a radio frequency (RF) plasma-enhanced chemical vapor deposition (PECVD) system. RF energy was inductively coupled into the deposition chamber through a quartz window. Special substrate treatment or catalysts were not required before deposition. The GP substrate was firstly cleaned with acetone and ethanol for several times, followed by drying in air, and then put on to the resistively heated sample stage that positioned a few centimeters below the quartz window. Methane (CH_4_) gas with a volume concentration range of 5%-100% in a H_2_ atmosphere was used as the carbon source for deposition. During the deposition process, the total gas flow rate was controlled at 5-10 sccm, and the gas pressure was kept at 6~12 Pa. The furnace temperature was set from 600 to 900°C. The as-received sample was cut into a square shape with a diameter of 1 cm as the electrode.

### 4.2. Electrochemical Measurements

CR2032 coin cells were assembled in an air-filled glovebox using VG@GP film as a working electrode and Li foil as a counter electrode for half-cell test. The Celgard 2500 was used as separator, and 1 M lithium bis (trifluoromethanesulfonyl) imide (LiTFSI) in 1,3-dioxolane (DOL) and 1,2 dimethoxyethane (DME) (1 : 1 *v*/*v*) with 1 wt% LiNO_3_ was employed as electrolyte. The cycling stability was carried out on a multichannel battery test system (Land 2001A Battery Testing System). For Coulombic efficiency test, certain amount of Li was deposited on VG@GP film electrode at different current densities and then stripped away to 1.0 V. To symmetric cell test, the VG@GP film electrode was firstly predeposited with 3 mAh cm^−2^ Li, and then the cell was discharged and charged at 1 mA cm^−2^ with a capacity of 1 mA cm^−2^. The electrolyte used for symmetrical cell test was 1 M LiTFSI in DOL/DME (1 : 1 *v*/*v*) with 1 wt% LiNO_3_, and the amount was 50 *μ*L. The electrochemical impedance spectroscopy (EIS) tests were performed on the PRASTAT P4000 electrochemical workstation with an amplitude of 5 mV over a frequency range of 10 mHz to 100 kHz. VMP3 electrochemical workstation was used to perform cyclic voltammetry (CV) tests in a voltage range of 0 to 3 V. For full cell test, LFP was used as the cathode material. The LFP powder, super P, and polyvinylidene fluoride (PVDF) were mixed in N-methyl-2-pyrrolidone (NMP) with a weight ratio of 8 : 1 : 1 and then cast onto an Al foil. The batteries with different mass loadings of LFP (3 and 20 mg cm^−2^) were tested. 1 M LiPF_6_ in ethylene carbonate (EC) : dimethyl carbonate (DMC) : ethyl methyl carbonate (EMC) (1 : 1:1 v/v) was used as the electrolyte, and the amount used was 40 and 50 *μ*L for cells with LFP loadings of 3 and 20 mg cm^−2^, respectively.

### 4.3. Characterization

The surface morphologies of VG@GP samples before and after Li deposition were probed by using a scanning electron microscope (SEM, HITACHI SU8010). Raman spectra were obtained by using a Horiba LabRAM HR800 with a 532 nm laser. The surface chemistry of samples was conducted by X-ray photoelectron spectroscopy (XPS) analyses on a PHI 5000 VersaProbe II spectrometer using monochromatic Al K(alpha) X-ray source.

## Supplementary Materials

Supplementary Materials Figure S1: optical image of 3D VG@GP film. Figure S2: XRD patterns of GP before and after discharging to 0V. Figure S3: cycling performance of 3D VG@GP and Cu foil electrodes at 1 mA cm^−2^ with a total capacity of 0.5 mAh cm^−2^. Figure S4: cycling performance of 3D VG@GP and Cu foil electrodes at 2 mA cm^−2^ with a total capacity of 1 mAh cm^−2^. Figure S5: cycling performance of 3D VG@GP and Cu foil electrodes at 5 mA cm^−2^ with a total capacity of 1 mAh cm^−2^. Figure S6: cycling performance of GP substrate at 1 mA cm^−2^ and 1 mAh cm^−2^. Figure S7: cycling performance of GP substrate at 2 mA cm^−2^ and 1 mAh cm^−2^. Figure S8: cycling performance of GP substrate at 3 mA cm^−2^ and 1 mAh cm^−2^. Figure S9: voltage profiles of Cu foil (a) and 3D VG@GP (b) and (c) the Li nucleation overpotentials on both electrodes at different current densities. Figure S10: discharge/charge curves of VG@GP. Figure S11: CV measurement of VG@GP. Figure S12: voltage profiles of Cu foil electrode at 1 mA cm^−2^ and 0.5 mAh cm^−2^. Figure S13: XPS spectra of VG@GP and Cu foil electrodes after 50 cycles: (a) C 1s spectra of VG@GP, (b) F 1s spectra of VG@GP, (c) C 1s spectra of Cu foil, and (d) F 1s spectra of Cu foil. Figure S14: the morphology of Li deposits after 50 cycles: (a) 3D VG@GP and (c) Cu foil. The morphology of Li deposits after 100 cycles: (b) 3D VG@GP and (d) Cu foil at 1 mA cm^−2^ with capacity of 0.5 mAh cm^−2^. The inset bar is 2.5 *μ*m. Figure S15: the surface morphology of Li deposits on GP substrate after (a) 50 cycles and (b) 100 cycles at 1 mA cm^−2^ with a capacity of 0.5 mAh cm^−2^. Figure S16: the surface morphology of Li deposits on GP substrate after (a) 50 cycles and (b) 100 cycles at 1 mA cm^−2^ with capacity of 1 mAh cm^−2^. Figure S17: the morphology of Li deposits after 50 cycles: (a) 3D VG@GP film and (b) Cu foil at 3 mA cm^−2^ with capacity of 1 mAh cm^−2^. Figure S18: the morphology of Li deposits after 25 cycles: (a) 3D VG@GP and (b) Cu foil at 1 mA cm^−2^ with capacity of 3 mAh cm^−2^. Figure S19: SEM image of the surface morphology (a) and cross-sectional structure (b) of VG@GP with a thickness of 5 *μ*m after cell assembly. Figure S20: the top view of surface morphology of Ni foam (a) before and (b) after cell assembly with the pressure. The cross-section view of surface morphology of Ni foam (c) before and (d) after cell assembly with the pressure. Figure S21: cycling performance of Ni foam at 3 mA cm^−2^ with a capacity of 1 mAh cm^−2^. Figure S22: voltage profiles of Li metal plating/stripping of 3D Li/VG@GP symmetric cell from 0.5 to 10 mA cm^−2^ with a capacity of 1 mAh cm^−2^ Figure S23: cycling performance of Li/VG@GP||LFP full cell at 0.5 C after 300 cycles. Figure 24: cycling performance of Li/VG@GP||LFP and Li/Cu||LFP full cells with a high LFP loading of 20 mg cm^−2^ at 0.3 C.
