# Supplementary material for: Vertical Graphenes Grown on a Flexible Graphite Paper as an All-Carbon Current Collector towards Stable Li Deposition
Source: Research (Wash D C). 2020 Jul 11;2020:7163948. doi: 10.34133/2020/7163948 (PMC7370242; doi:10.34133/2020/7163948)
Supplement: Supplementary Materials — Figure S1: optical image of 3D VG@GP film. Figure S2: XRD patterns of GP before and after discharging to 0V. Figure S3: cycling performance of 3D VG@GP and Cu foil electrodes at 1 mA cm−2 with a total capacity of 0.5 mAh cm−2. Figure S4: cycling performance of 3D VG@GP and Cu foil electrodes at 2 mA cm−2 with a total capacity of 1 mAh cm−2. Figure S5: cycling performance of 3D VG@GP and Cu foil electrodes at 5 mA cm−2 with a total capacity of 1 mAh cm−2. Figure S6: cycling performance of GP substrate at 1 mA cm−2 and 1 mAh cm−2. Figure S7: cycling performance of GP substrate at 2 mA cm−2 and 1 mAh cm−2. Figure S8: cycling performance of GP substrate at 3 mA cm−2 and 1 mAh cm−2. Figure S9: voltage profiles of Cu foil (a) and 3D VG@GP (b) and (c) the Li nucleation overpotentials on both electrodes at different current densities. Figure S10: discharge/charge curves of VG@GP. Figure S11: CV measurement of VG@GP. Figure S12: voltage profiles of Cu foil electrode at 1 mA cm−2 and 0.5 mAh cm−2. Figure S13: XPS spectra of VG@GP and Cu foil electrodes after 50 cycles: (a) C 1s spectra of VG@GP, (b) F 1s spectra of VG@GP, (c) C 1s spectra of Cu foil, and (d) F 1s spectra of Cu foil. Figure S14: the morphology of Li deposits after 50 cycles: (a) 3D VG@GP and (c) Cu foil. The morphology of Li deposits after 100 cycles: (b) 3D VG@GP and (d) Cu foil at 1 mA cm−2 with capacity of 0.5 mAh cm−2. The inset bar is 2.5 μm. Figure S15: the surface morphology of Li deposits on GP substrate after (a) 50 cycles and (b) 100 cycles at 1 mA cm−2 with a capacity of 0.5 mAh cm−2. Figure S16: the surface morphology of Li deposits on GP substrate after (a) 50 cycles and (b) 100 cycles at 1 mA cm−2 with capacity of 1 mAh cm−2. Figure S17: the morphology of Li deposits after 50 cycles: (a) 3D VG@GP film and (b) Cu foil at 3 mA cm−2 with capacity of 1 mAh cm−2. Figure S18: the morphology of Li deposits after 25 cycles: (a) 3D VG@GP and (b) Cu foil at 1 mA cm−2 with capacity of 3 mAh cm−2. Figure S19 [file 7163948.f1.doc]

Supporting Information

Vertical graphenes grown on a flexible graphite paper as an all-carbon current collector towards stable Li deposition

Zhijia Huang^†^, Debin Kong^†^, Yunbo Zhang, Yaqian Deng, Guangmin Zhou*, Chen Zhang, Feiyu Kang, Wei Lv*, Quan-Hong Yang*

**Supporting Figures and Texts**


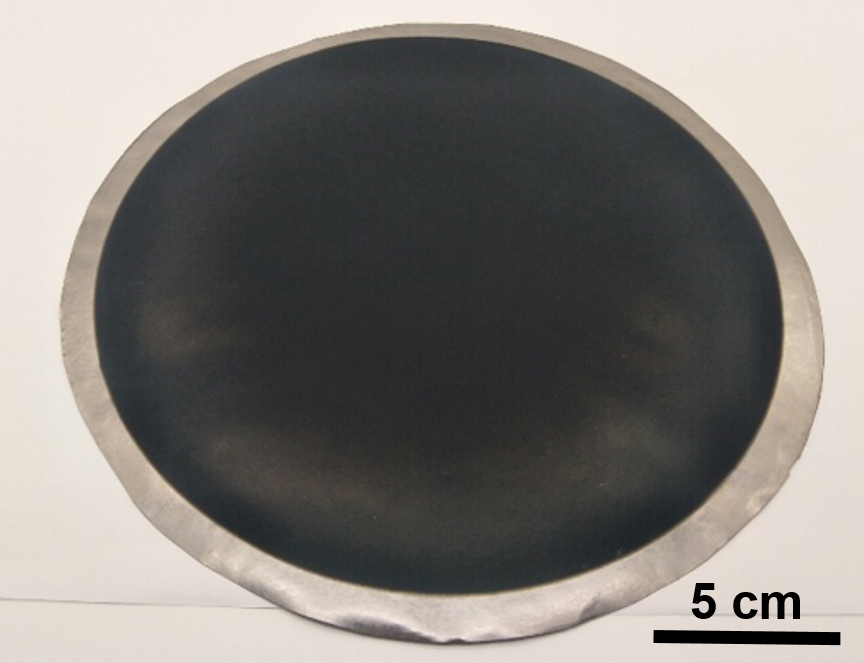


**Figure S1.** Optical image of 3D VG@GP film.


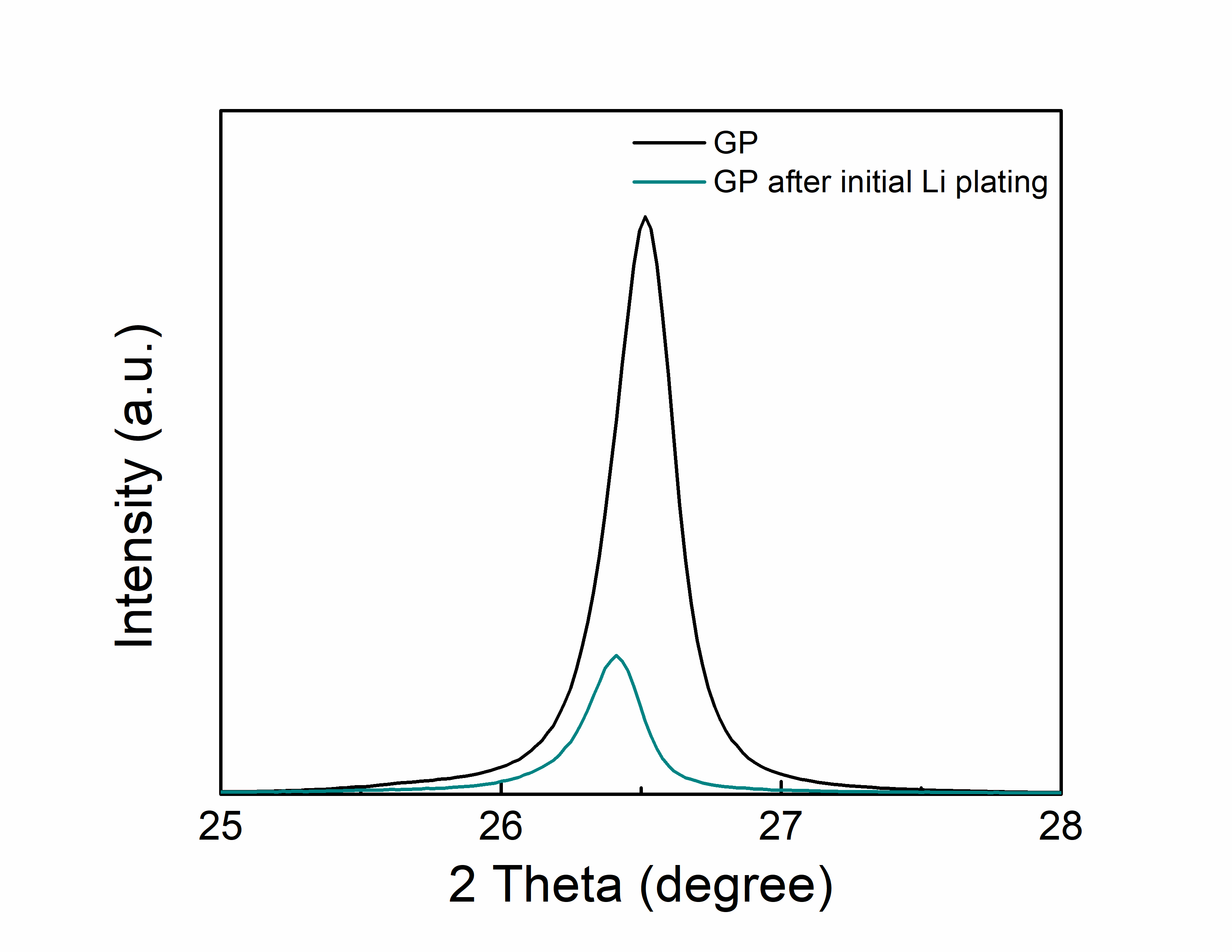


**Figure S2.** XRD patterns of GP before and after discharging to 0 V.


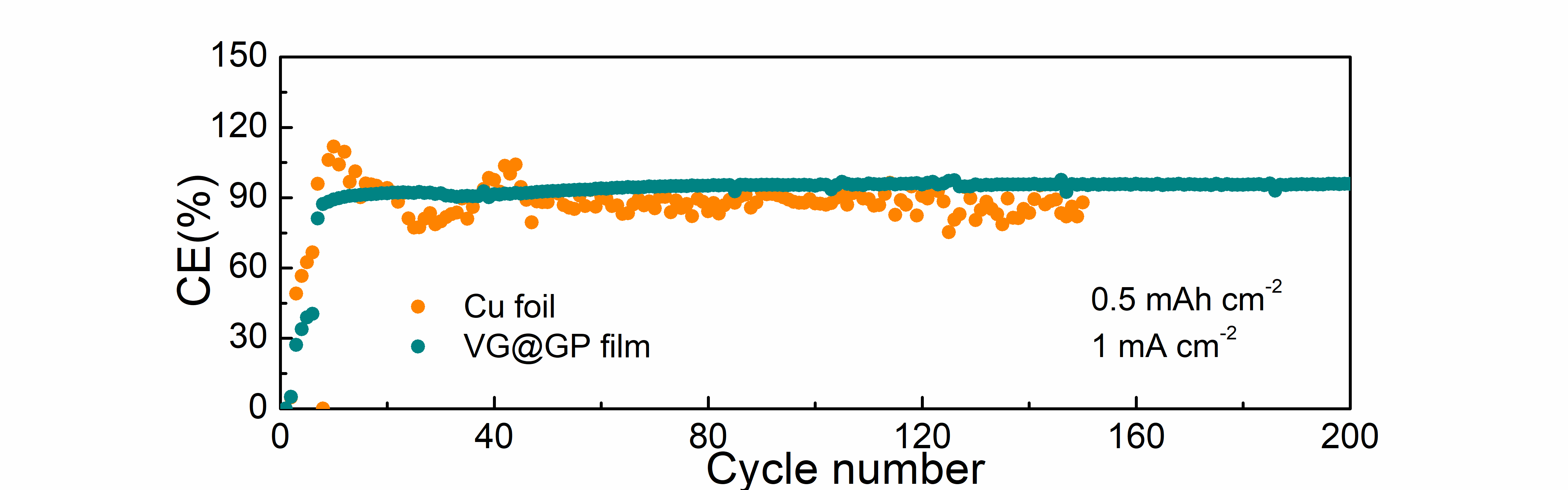


**Figure S3.** Cycling performance of 3D VG@GP and Cu foil electrodes at 1 mA cm^-2^ with a total capacity of 0.5 mAh cm^-2^.


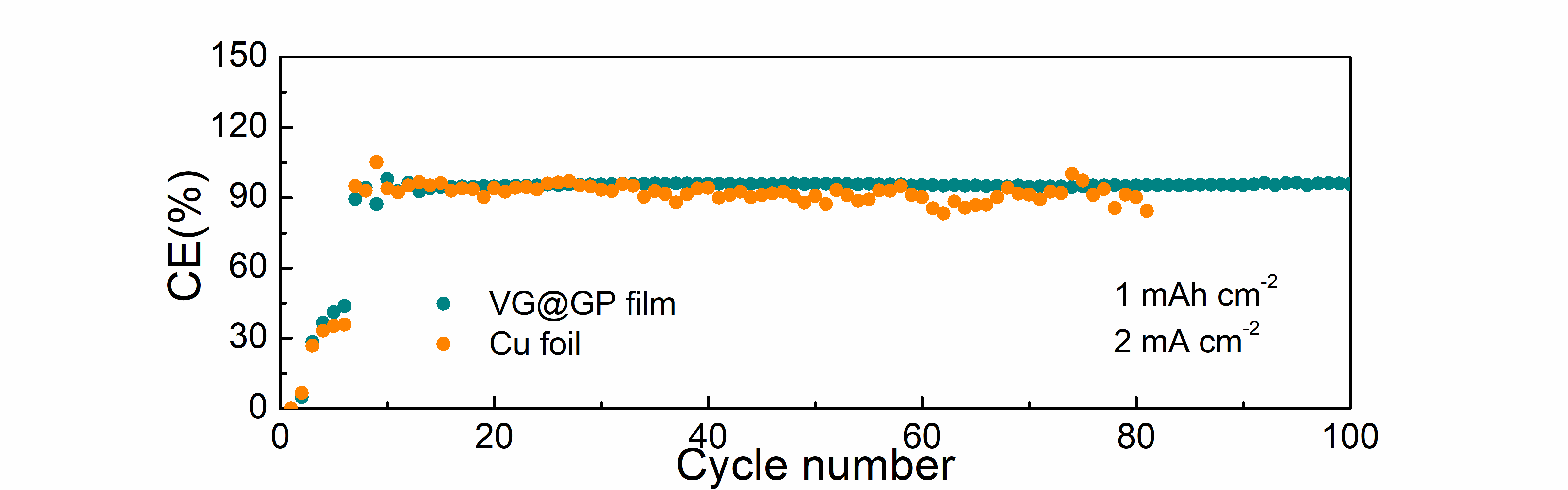


**Figure S4.** Cycling performance of 3D VG@GP and Cu foil electrodes at 2 mA cm^-2^ with a total capacity of 1 mAh cm^-2^.


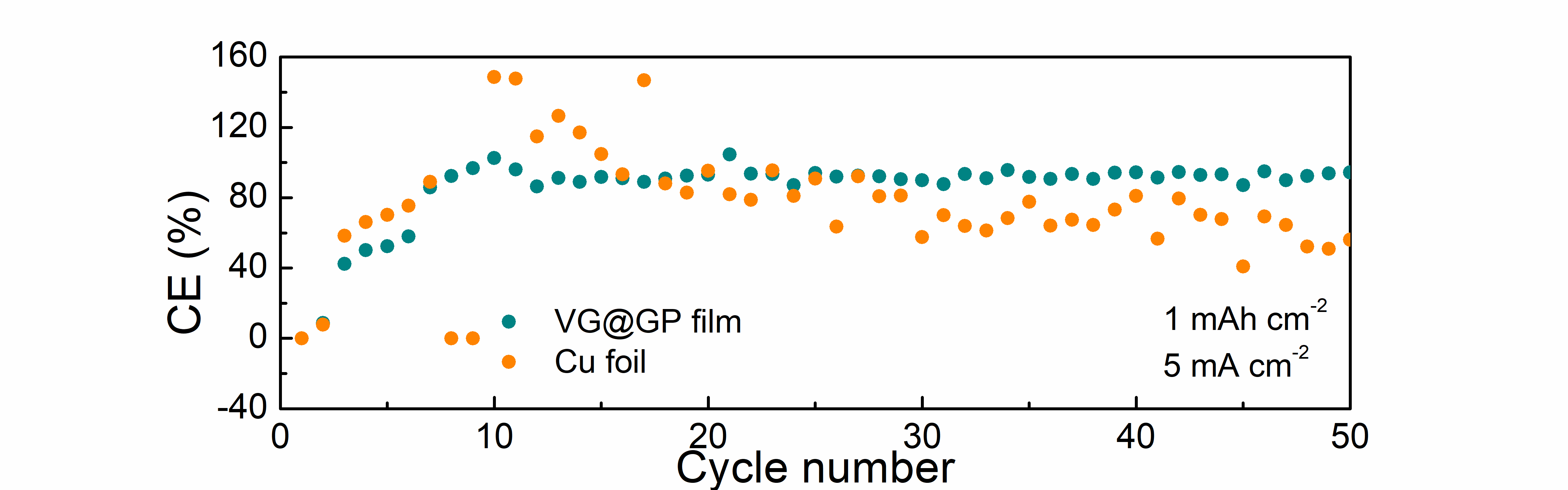


**Figure S5.** Cycling performance of 3D VG@GP and Cu foil electrodes at 5 mA cm^-2^ with a total capacity of 1 mAh cm^-2^.


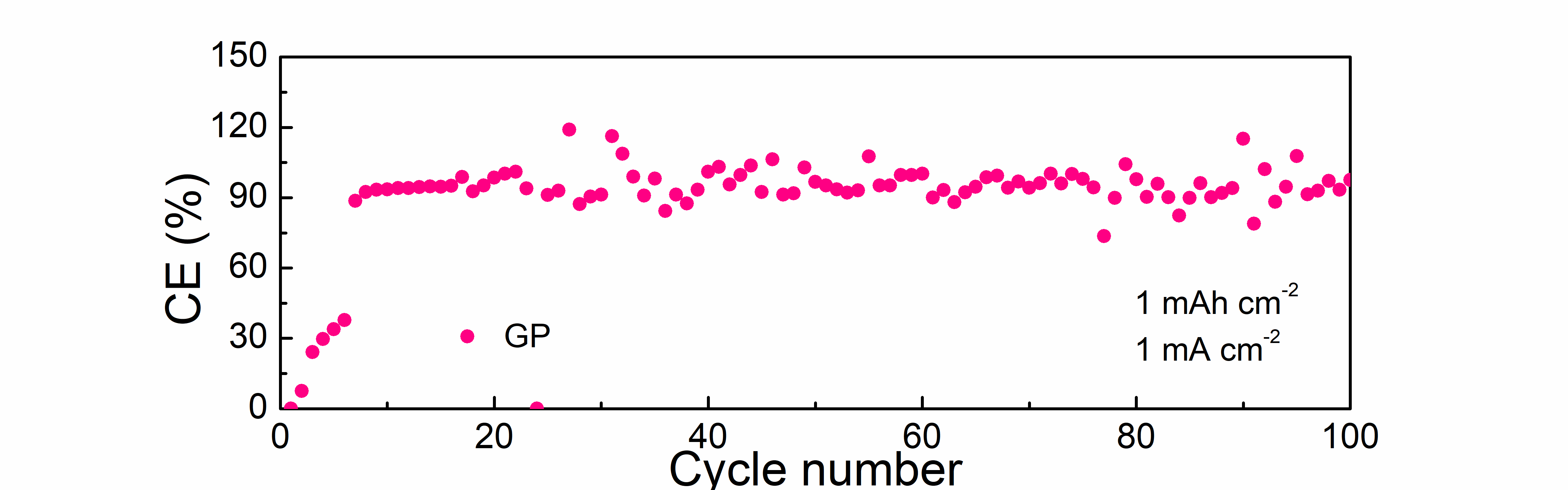


**Figure S6.** Cycling performance of GP substrate at 1 mA cm^-2^ and 1 mAh cm^-2^.


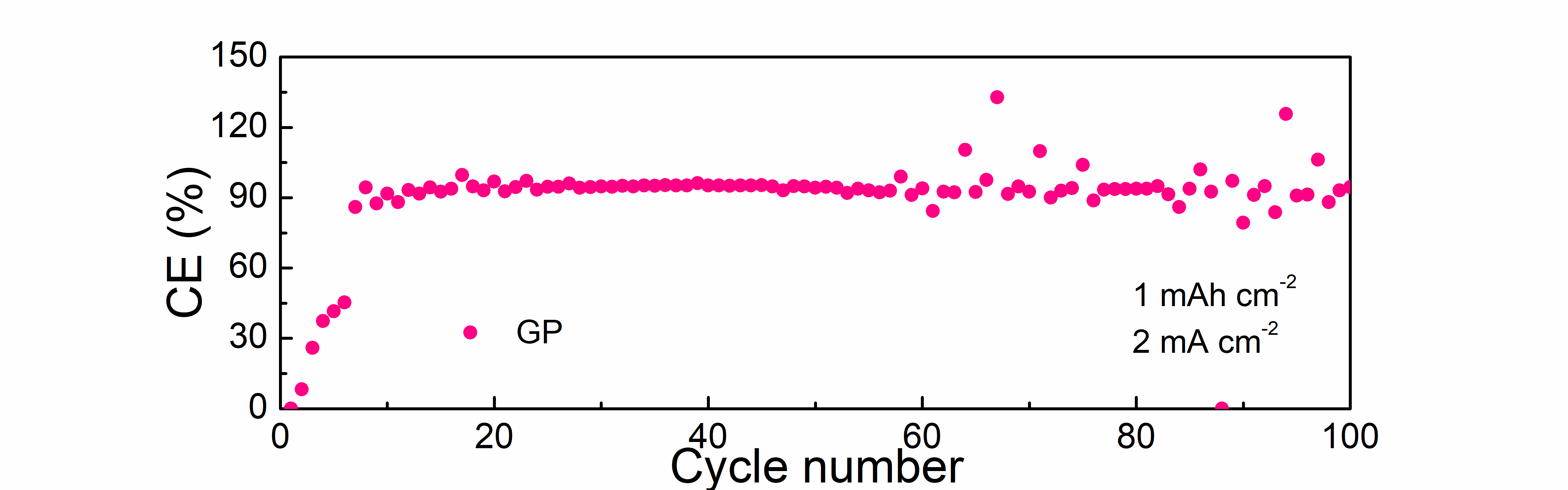


**Figure S7.** Cycling performance of GP substrate at 2 mA cm^-2^ and 1 mAh cm^-2^.


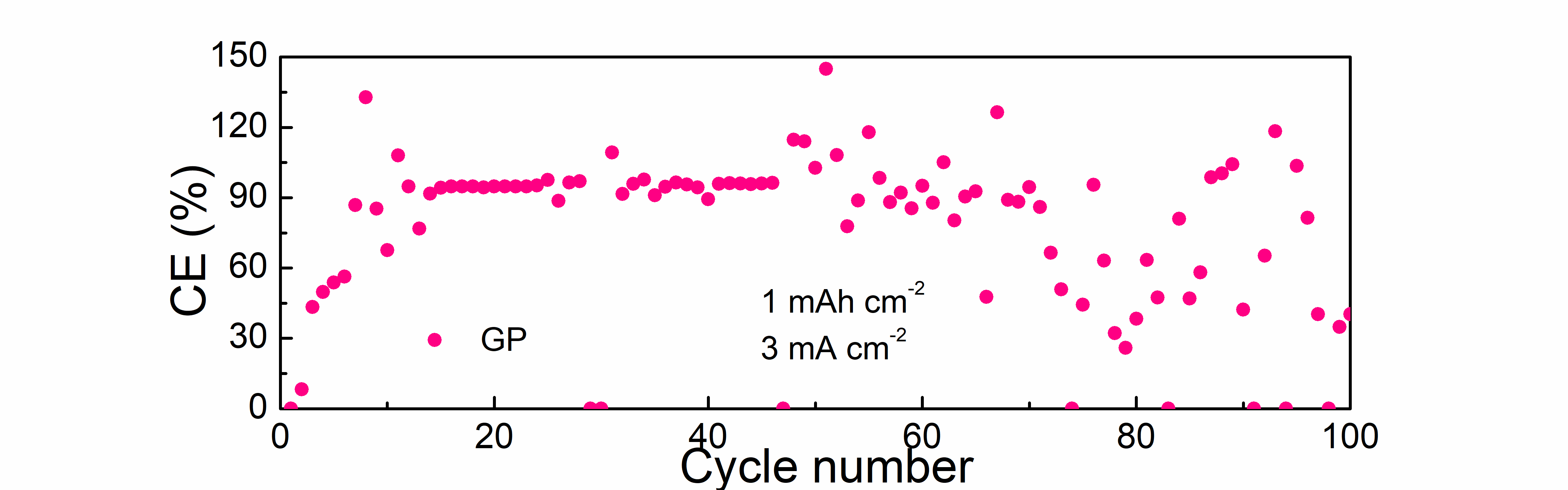


**Figure S8.** Cycling performance of GP substrate at 3 mA cm^-2^ and 1 mAh cm^-2^.


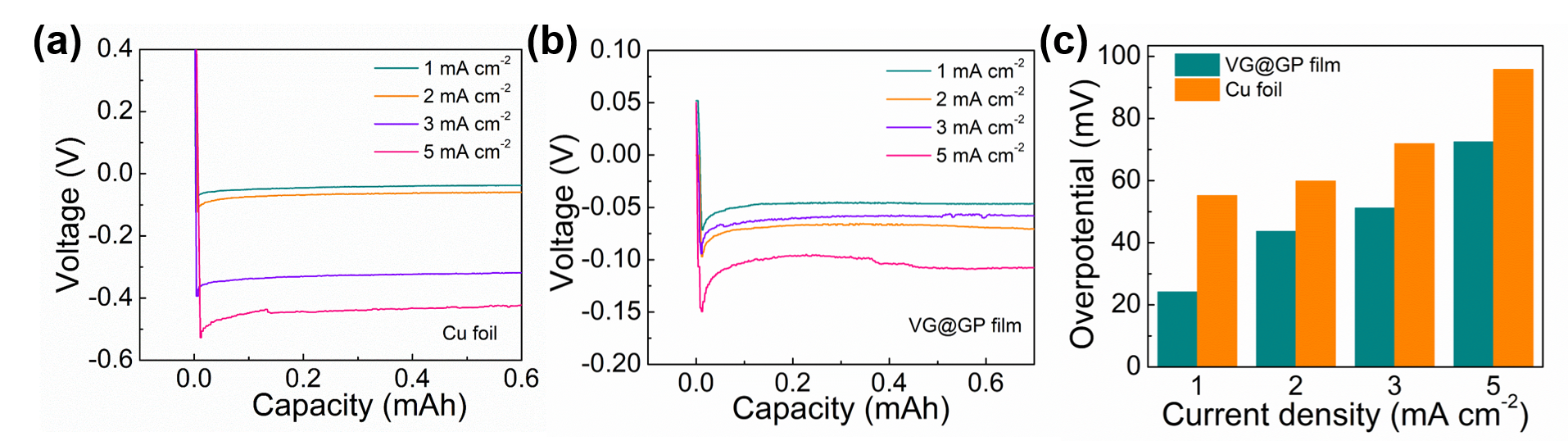


**Figure S9.** Voltage profiles of Cu foil (a) and 3D VG@GP (b) and (c) the Li nucleation overpotentials on both electrodes at different current densities.


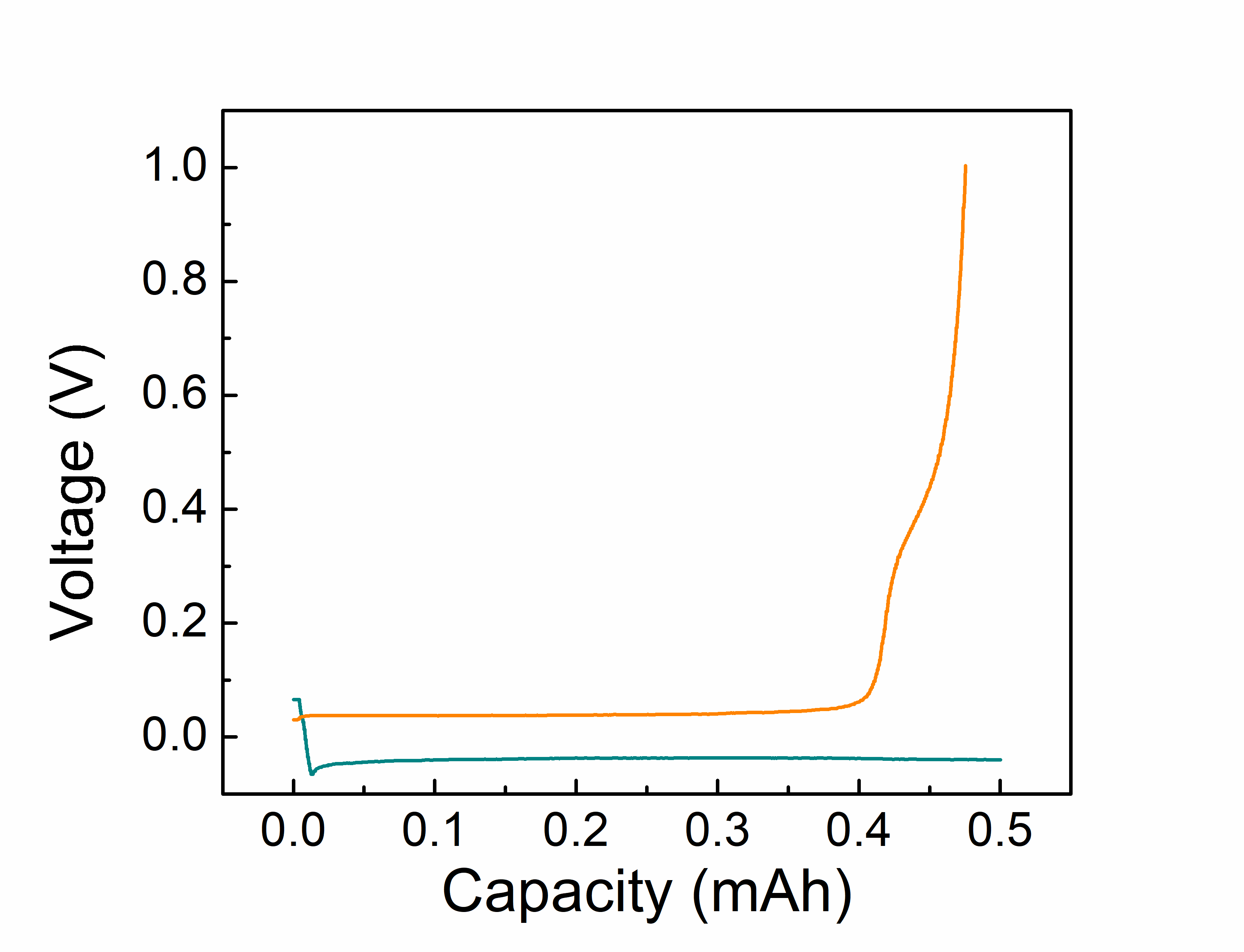


**Figure S10.** Discharge/charge curves of VG@GP.


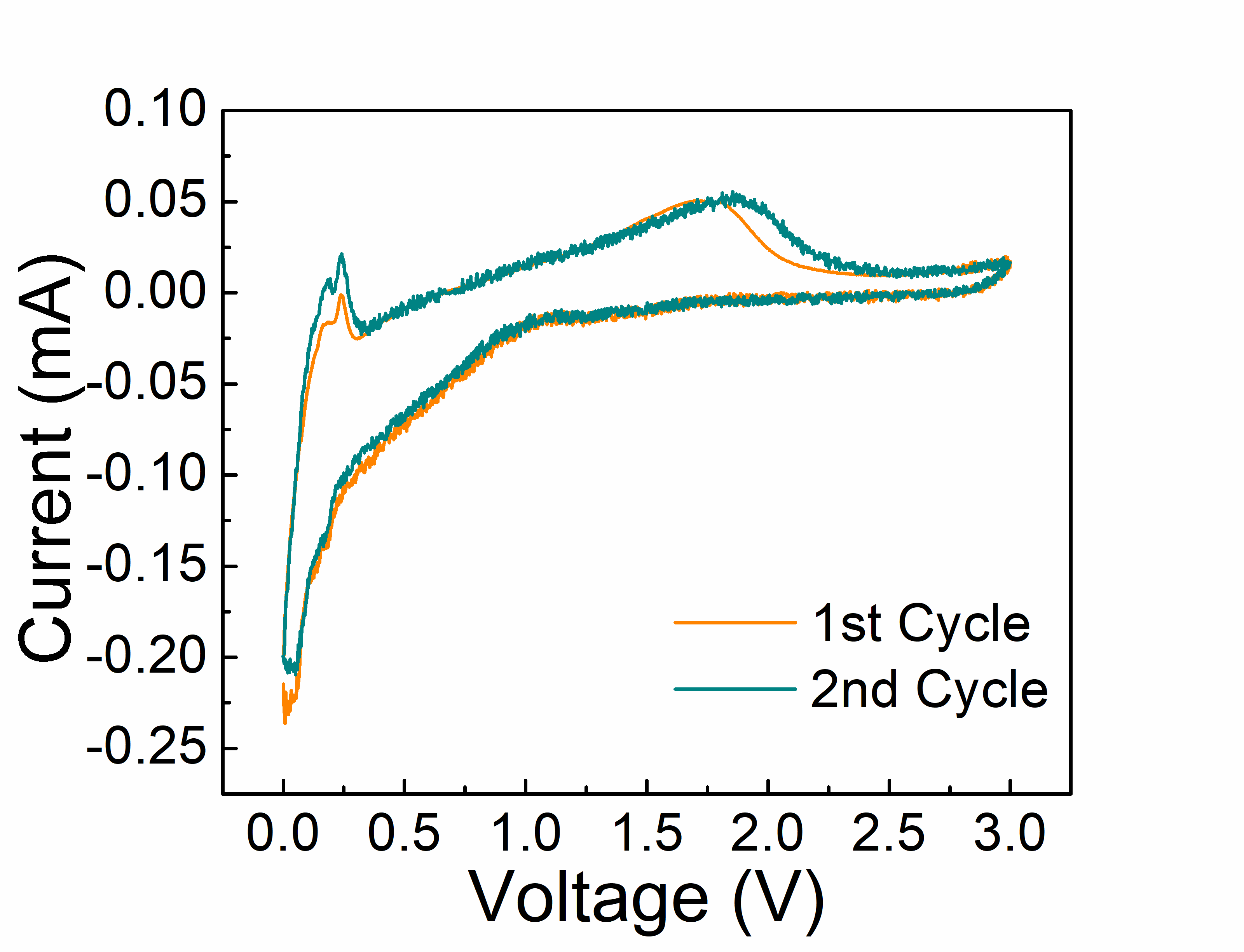


**Figure S11.** CV measurement of VG@GP.

**
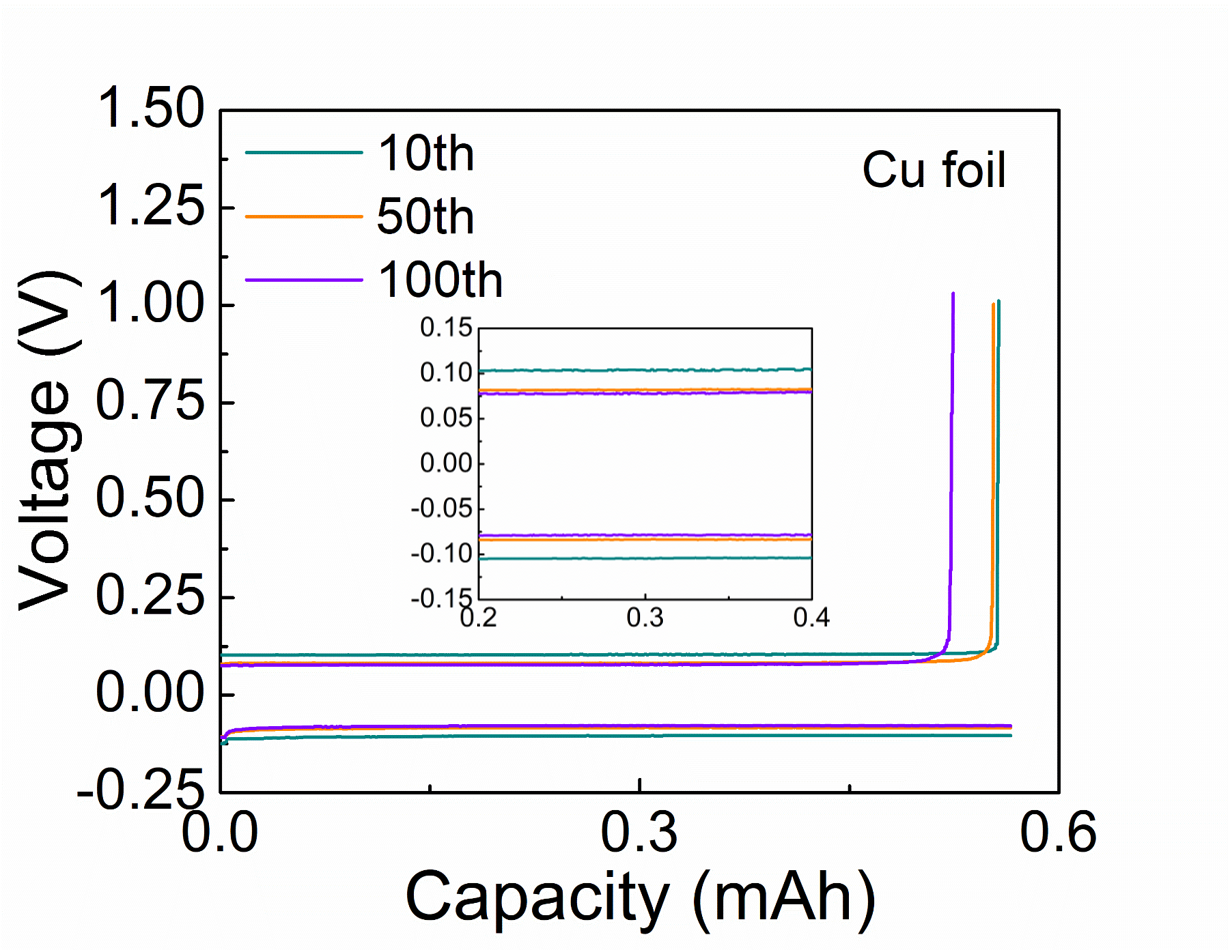
**

**Figure S12.** Voltage profiles of Cu foil electrode at 1 mA cm^-2^ and 0.5 mAh cm^-2^.


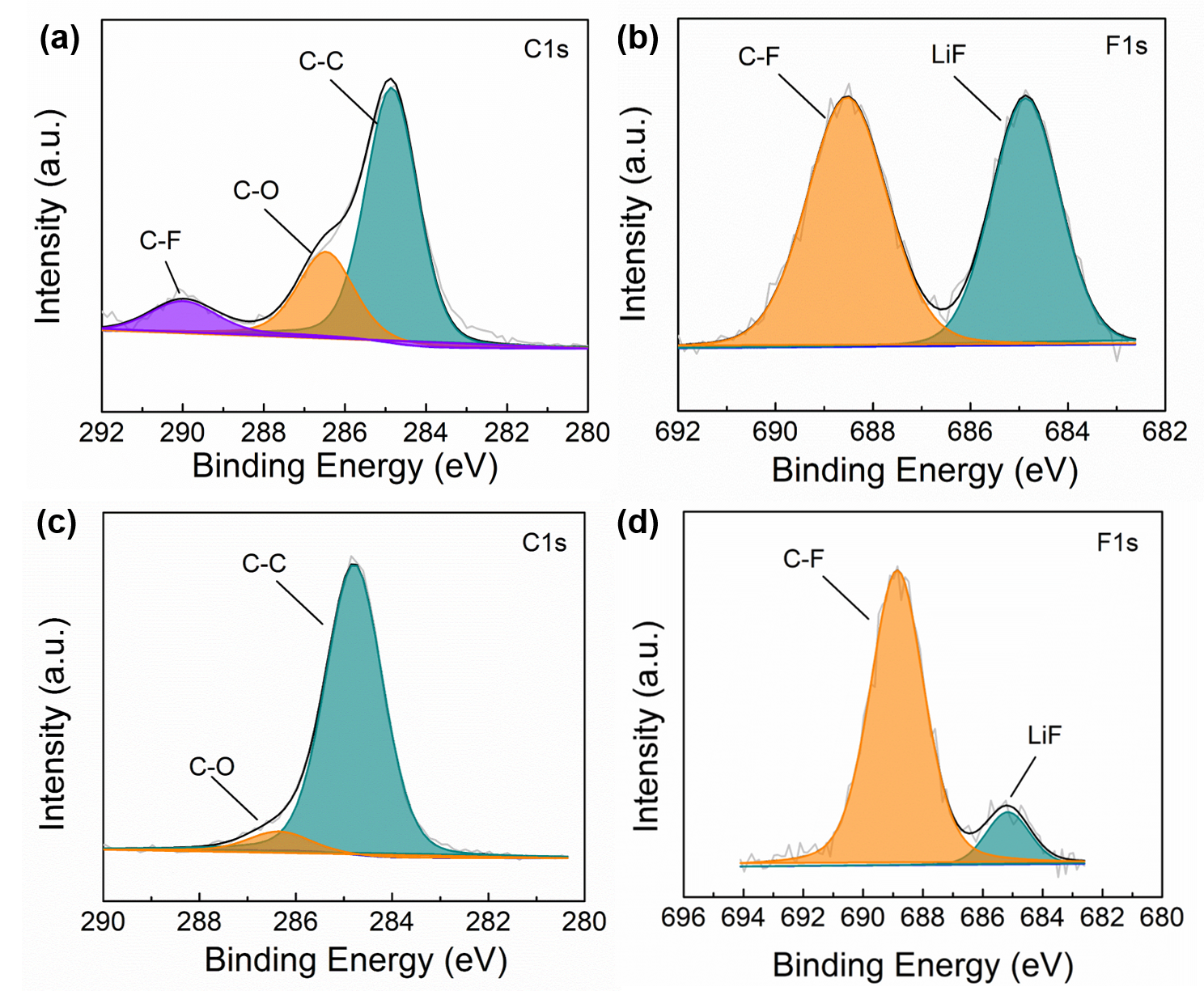


**Figure S13**. XPS spectra of VG@GP and Cu foil electrodes after 50 cycles: (a) C 1s spectra of VG@GP; (b) F 1s spectra of VG@GP; (c) C 1s spectra of Cu foil and (d) F 1s spectra of Cu foil.


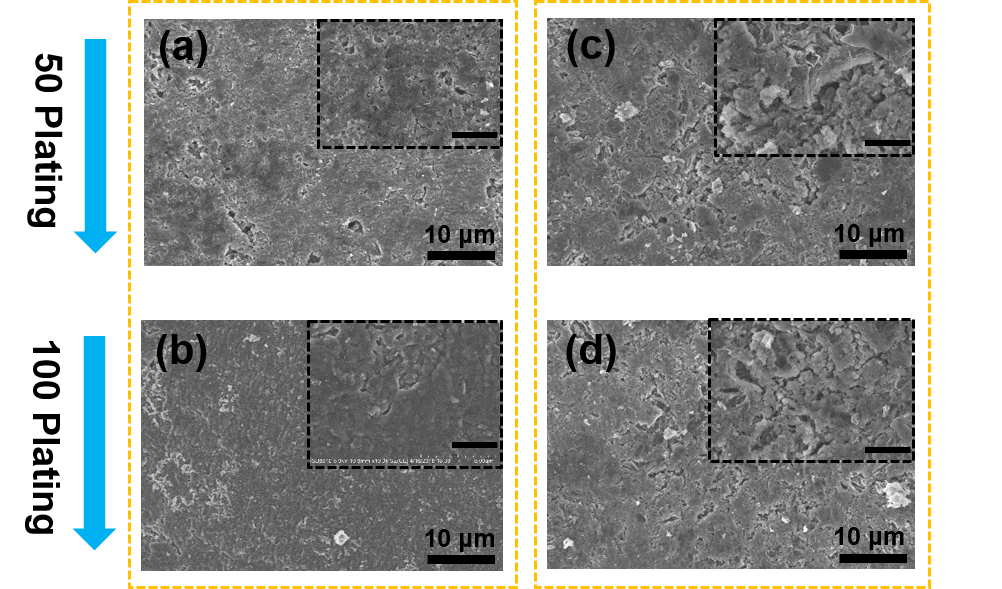


**Figure S14.** The morphology of Li deposits after 50 cycles: (a) 3D VG@GP and (c) Cu foil. The morphology of Li deposits after 100 cycles: (b) 3D VG@GP and (d) Cu foil at 1 mA cm^-2^ with capacity of 0.5 mAh cm^-2^. The inset bar is 2.5 μm.


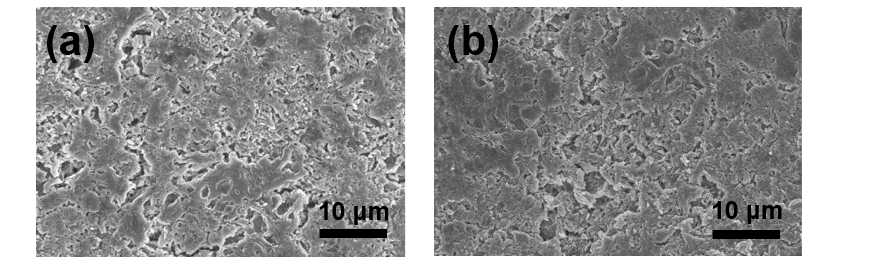


**Figure S15.** The surface morphology of Li deposits on GP substrate after (a) 50 cycles and (b) 100 cycles at 1 mA cm^-2^ with a capacity of 0.5 mAh cm^-2^.


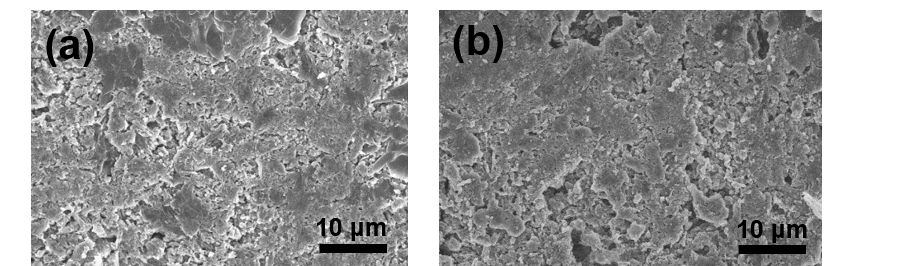


**Figure S16.** The surface morphology of Li deposits on GP substrate after (a) 50 cycles and (b) 100 cycles at 1 mA cm^-2^ with capacity of 1 mAh cm^-2^.


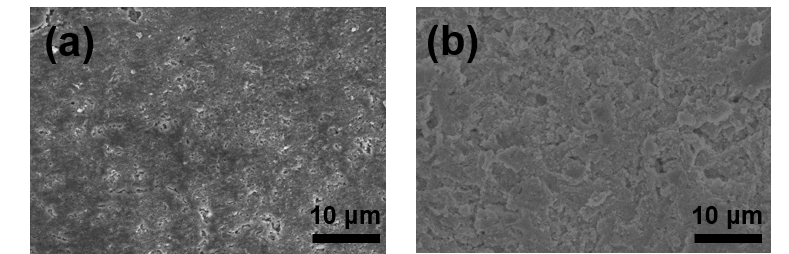


**Figure S17.** The morphology of Li deposits after 50 cycles: (a) 3D VG@GP film and (b) Cu foil at 3 mA cm^-2^ with capacity of 1 mAh cm^-2^.


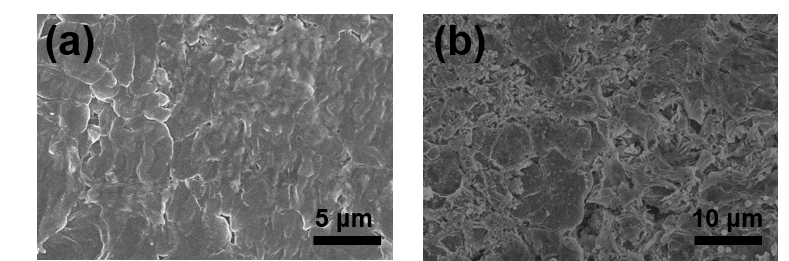


**Figure S18.** The morphology of Li deposits after 25 cycles: (a) 3D VG@GP and (b) Cu foil at 1 mA cm^-2^ with capacity of 3 mAh cm^-2^.


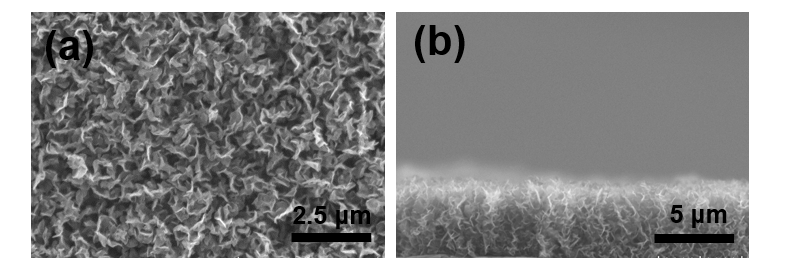


**Figure S19.** SEM image of the surface morphology (a) and cross-sectional structure (b) of VG@GP with a thickness of 5 µm after cell assembly.


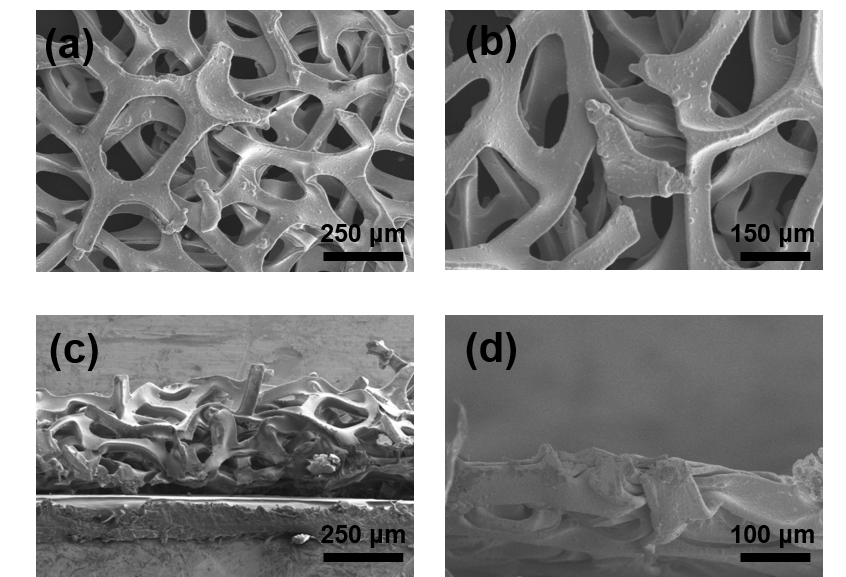


**Figure S20.** The top view of surface morphology of Ni foam (a) before and (b) after cell assembly with the pressure. The cross-section view of surface morphology of Ni foam (c) before and (d) after cell assembly with the pressure.


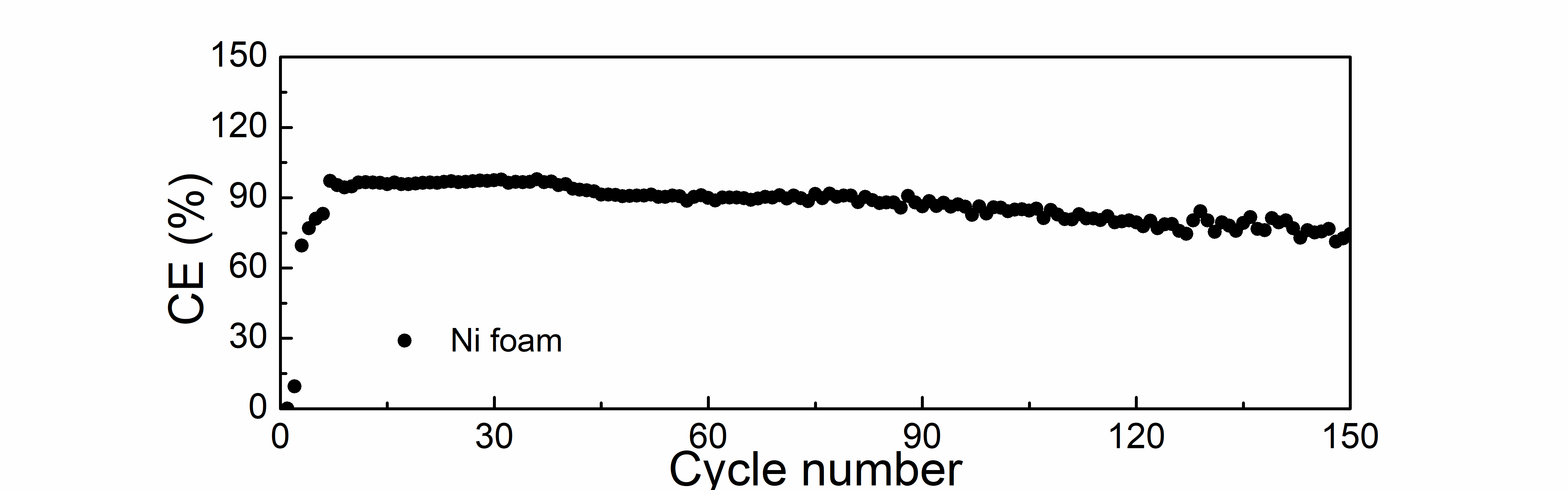


**Figure S21.** Cycling performance of Ni foam at 3 mA cm^-2^ with a capacity of 1 mAh cm^-2^.


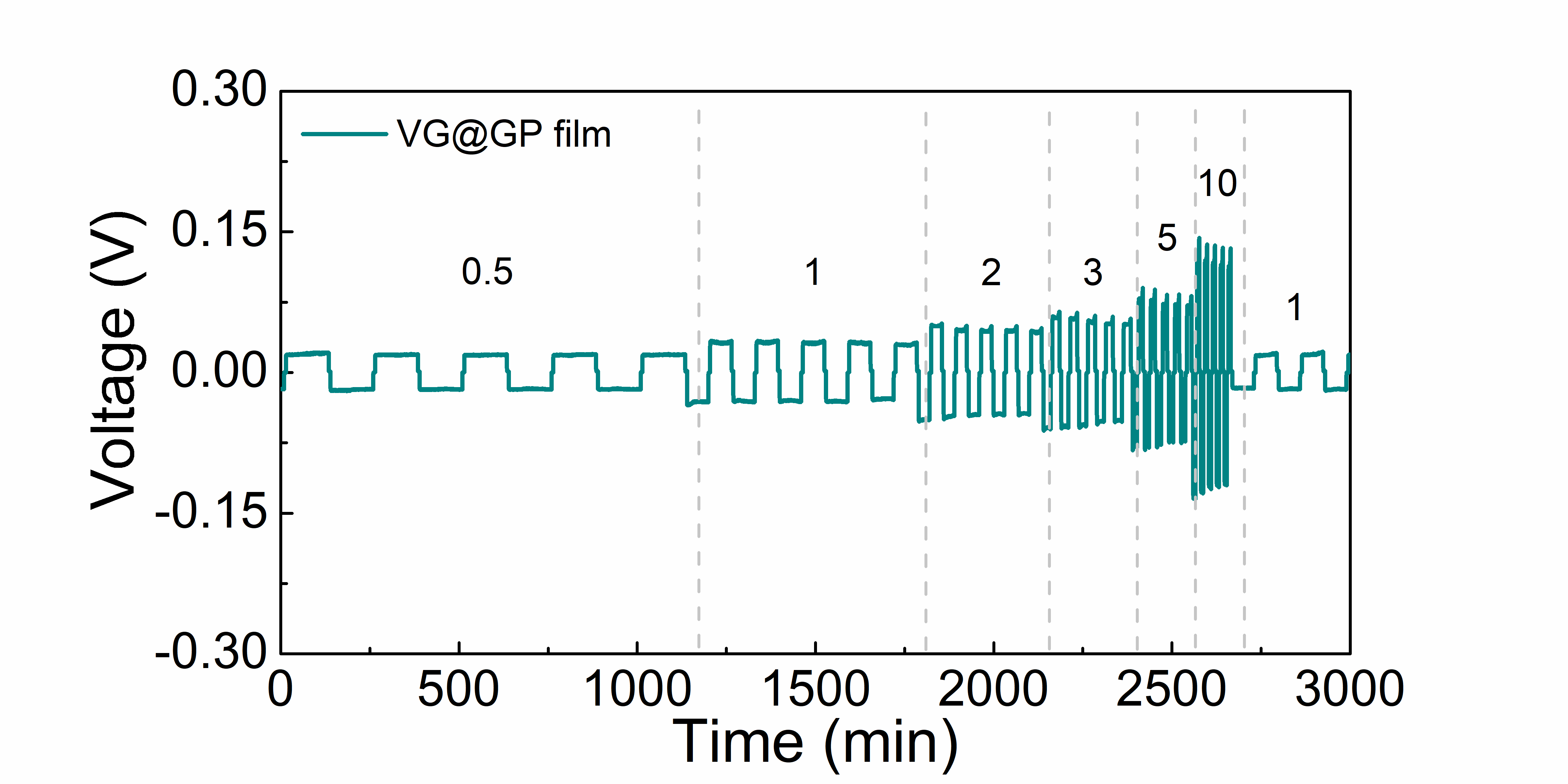


**Figure S22.** Voltage profiles of Li metal plating/stripping of 3D Li/VG@GP symmetric cell from 0.5 to 10 mA cm^-2^ with a capacity of 1 mAh cm^-2^.


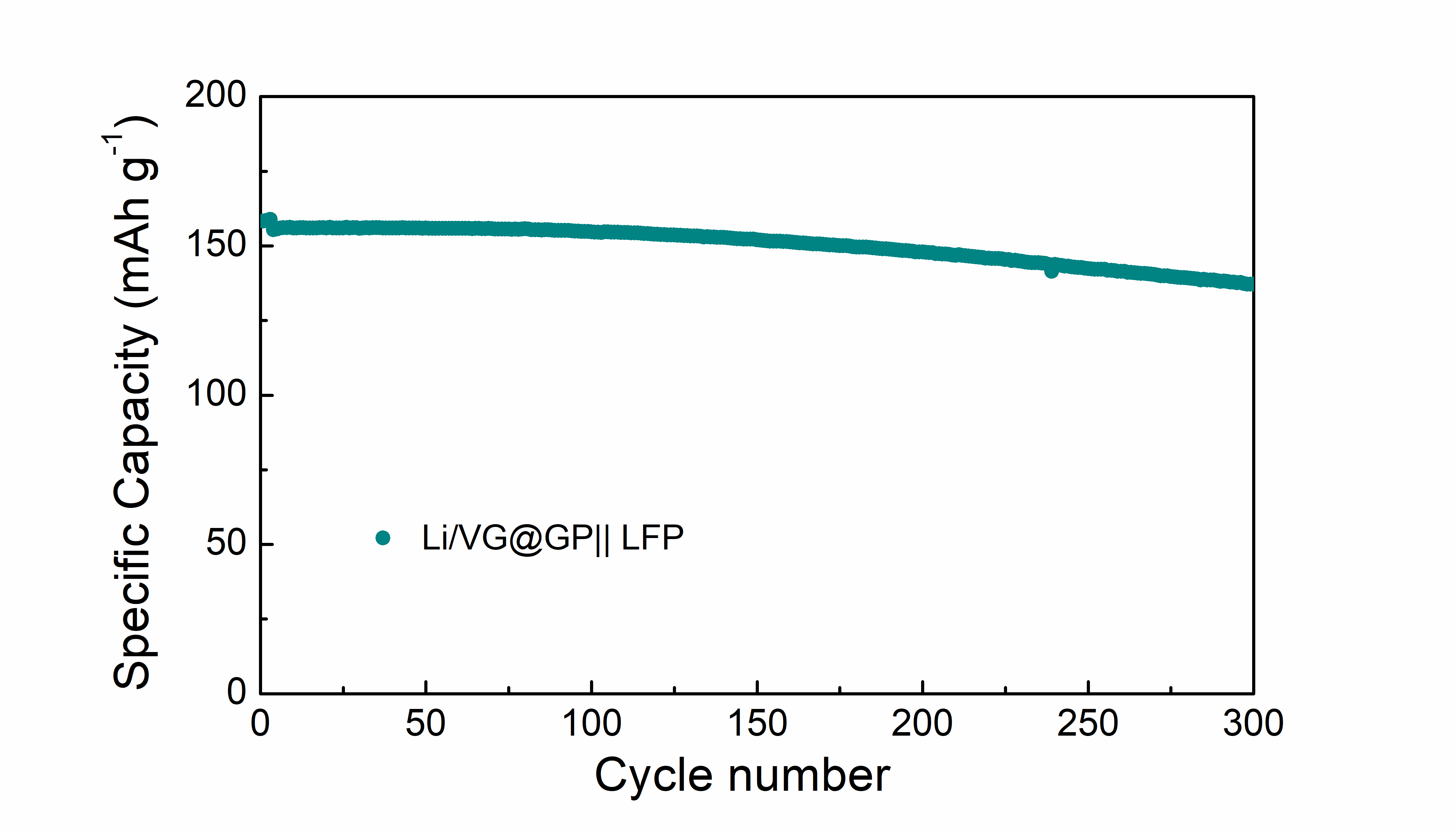


**Figure S23.** Cycling performance of Li/VG@GP||LFP full cell at 0.5 C after 300 cycles.


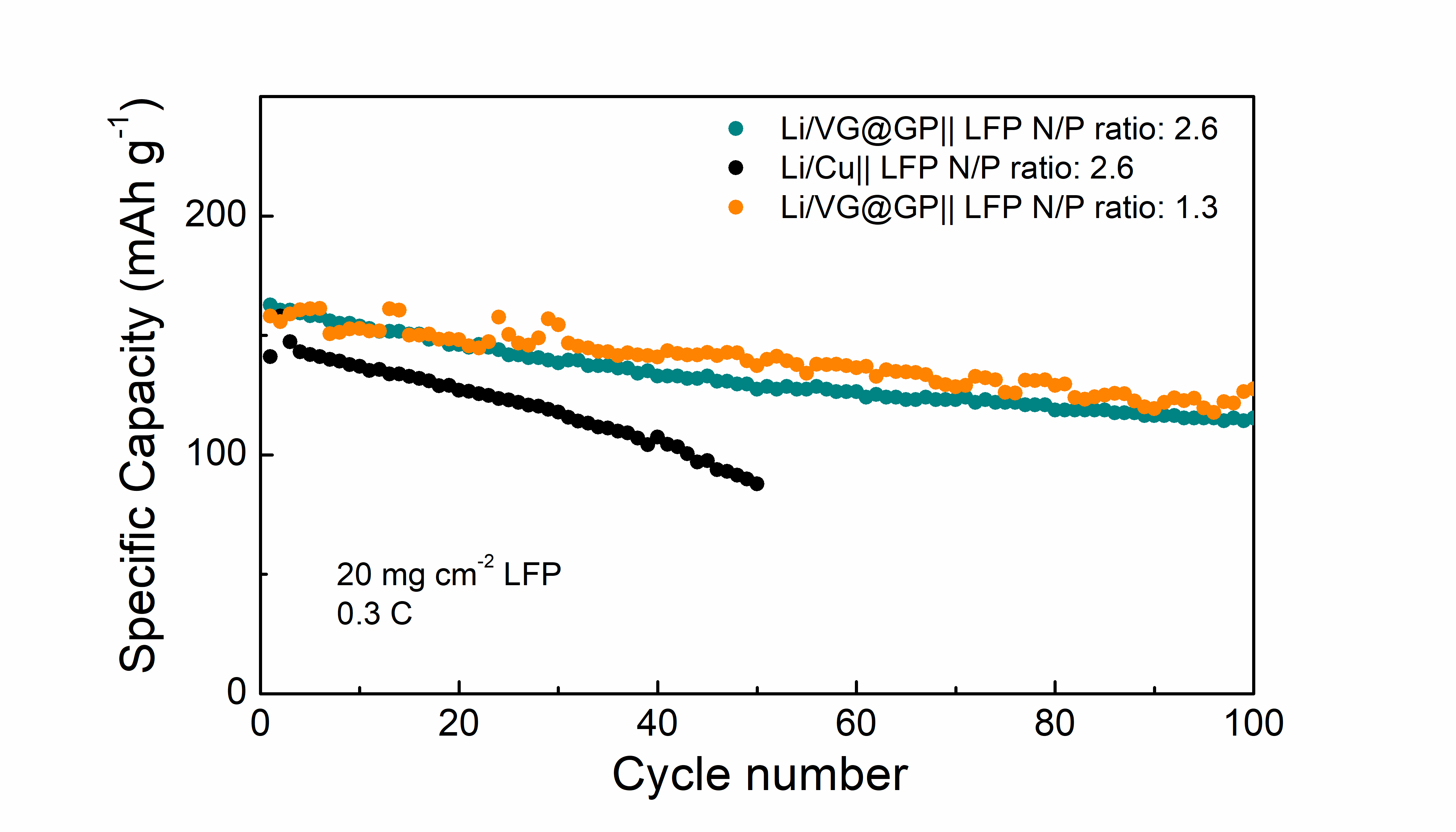


**Figure 24.** Cycling performance of Li/VG@GP||LFP and Li/Cu||LFP full cells with a high LFP loading of 20 mg cm^-2^ at 0.3 C.
